# Fostering inclusion in EEG measures of pediatric brain activity

**DOI:** 10.1038/s41539-024-00240-y

**Published:** 2024-04-02

**Authors:** Eryn J. Adams, Molly E. Scott, Melina Amarante, Chanel A. Ramírez, Stephanie J. Rowley, Kimberly G. Noble, Sonya V. Troller-Renfree

**Affiliations:** 1 Department of Psychology, University of New Orleans, New Orleans, LA 70148 USA; 2 Department of Biobehavioral Sciences, Teachers College, Columbia University, New York, NY 10027 USA; 3 School of Education and Human Development, University of Virginia, Charlottesville, VA USA; 4 Department of Human Development, Teachers College, Columbia University, New York, NY 10027 USA

**Keywords:** Human behaviour, Education

## Abstract

The past two decades have seen a rapid increase in neuroscientific evidence being used to characterize how contextual, structural, and societal factors shape cognition and school readiness. Measures of functional brain activity are increasingly viewed as markers of child development and biomarkers that could be employed to track the impact of interventions. While electroencephalography (EEG) provides a promising tool to understand educational inequities, traditional EEG data acquisition is commonly limited in some racial and ethnic groups due to hair types and styles. This ultimately constitutes unintentional systemic racism by disproportionately excluding participants from certain racial and ethnic groups from participation and representation in neuroscience research. Here, we provide a comprehensive review of how cultural considerations surrounding hair density, texture, and styling consistently skew samples to be less representative by disproportionately excluding Black and Latinx participants. We also provide recommendations and materials to promote best practices.

The past two decades have seen a rapid increase in neuroscientific research^1,2^, with neuroscientific evidence becoming increasingly influential in education and policy settings. Neuroscientific measures have been used to characterize normative development in diverse participants and how contextual, structural, and societal factors shape cognition and school readiness^3–5^. Furthermore, neuroscientific measures have been posited as an outcome targeted for change by interventions as well as a way to assess the impact of interventions designed to reduce inequality and adversity^6–9^. While neuroscience is a promising tool for understanding and characterizing educational inequalities, it is important to acknowledge known racial and ethnic biases in neuroscientific data, including overrepresentation of White participants, disproportional exclusion of non-white participants due to phenotypic differences (e.g., hair texture or skin color), as well as ignorance of lived experiences and racism in neuroscientific research^10–12^.

Pediatric electroencephalography (EEG) is a robust neuroscientific tool that directly records neural activity in children. To collect EEG, small recording devices called electrodes make contact with the scalp to record the brain’s electrical activity. EEG has numerous benefits, including greater sensitivity than behavioral measures; substantially lower costs than other neuroimaging techniques (e.g., fMRI); robustness to demand characteristics; the ability to record the same outcome across the lifespan; the ability to record in non-lab settings^13^; and relatively good data quality in the face of movement, which allows participants to be awake and moving during recordings, even during infancy. The past two decades have seen an exponential increase in developmental EEG research, including implementation in large-scale projects like Environmental Influences on Child Health Outcomes (ECHO)^14^ and HEALthy Brain and Child Development Study (HBCD)^15^.

However, as EEG research expands, it is important to note that traditional methods of EEG data acquisition with standard equipment is often limited in some racial and ethnic groups due to common hair types and styles. This ultimately constitutes unintentional systemic racism, by disproportionately excluding participants from certain racial and ethnic groups from participation and representation in neuroscience research^10,16^. Thick, voluminous, and curly hair types and certain hairstyles can hinder researchers’ attempts to collect high-quality EEG data, ultimately leading to the disproportionate exclusion of minority populations from research. Such exclusion leads to reductions in the diversity of the participants from which we acquire EEG data and limits the generalizability of research findings^10,16^. This practice may, consequently, misinform interventions aimed at reducing educational inequalities.

Given the increasing popularity of EEG research among pediatric populations – and a growing aim of this research to inform educational interventions and policies – it is essential for researchers to minimize unintentional systemic racism and race-related biases in this work. The present paper aims to share materials, experiences, and recommendations from a diverse team of researchers who have collected more than 2000 pediatric EEGs from families who mostly identify as Black and/or Latinx over the last five years (see a more complete discussion of racial and ethnic categories in *The importance of hair for inclusivity in EEG*). In doing so, we hope to empower fellow pediatric EEG researchers, and to begin reducing systemic racism stemming from disproportionate participant exclusion in current pediatric EEG research.

In pursuit of this goal, we have organized the present paper to reflect the natural order of a pediatric EEG research visit. First, we begin by discussing why understanding cultural differences in hair is important for inclusivity in EEG (*The importance of hair for inclusivity in EE*G). Next, in the section entitled *Preparing families for a pediatric EEG recording*, we take what we have learned from the literature and our conversations with participants, researchers with lived experience, and community partners to provide direct recommendations and resources for preparing families for an EEG. These recommendations span all research activities before conducting the EEG recording including scheduling communications, lab arrival, consent, and assent. Following participant preparation recommendations, we discuss how to prepare and select the recording equipment most likely to result in successful cap application and high-quality data collection in *Conducting a pediatric EEG lab visit*. In the section *Considerations for capping different hair textures and styles*, we provide specific recommendations for capping a variety of different common hair textures and styles seen in pediatric samples, including scalp braids, plaits, braids, twists, loose and natural styles, puffs, ponytails, and locs. Finally, we wrap up with *Recommendations for future pediatric EEG research* and general *Conclusions*.

## The importance of hair for inclusivity in EEG

One of the largest issues facing diversity and generalizability in EEG research is practical – most EEG system hardware is not designed to work well with dense, curly, and/or coily hair types^10,16–18^. While many of these issues stem from technical limitations in the hardware (see the section on *Equipment Considerations* below), another source of potential bias in pediatric EEG stems from cultural preferences and routines surrounding hair texture, washing, and styling. In particular, participants who identify as Black and Latinx are more likely to have hair textures and styles that make EEG recordings more difficult.

Before discussing specific considerations surrounding hair, we note that cultural groups are not monolithic. For example, Black participants who identify as African American may have different preferences than participants who identify as Caribbean American. Similar distinctions can be drawn among those who identify as Latinx, as Latinx is an ethnic category that is comprised of individuals who identify with multiple geographic and racial backgrounds. There is currently an active discussion on how to refer to the Latinx community inclusively. Here we chose ‘Latin’ over ‘Hispanic’ as our research team has almost exclusively conducted research with participants from Latin American countries. Furthermore, we chose to use ‘Latinx’ as it is gender neutral and it was agreed upon by members of our authorship team who identify as Latina. However, we understand that members of our research staff and participant community prefer other terms such as Latino, Latina, Latin@, Latine, or Hispanic and we try to honor those preferences where possible. We also note that some participants identify as both Black and Latinx and that insights and recommendations from both the Black and Latinx sections may apply to these individuals. That said, we acknowledge that some members of the Latinx Additionally, racial and/or ethnic identity can be complicated, with some participants identifying as multiracial or as not belonging to a single racial and/or ethnic identity; this can further complicate conversations surrounding EEG and hair^19^. These complexities are exacerbated when families do not identify with certain racial and/or ethnic categories often used by researchers (e.g., census-based categories). It is important to recognize that the below observations, conversations, and recommendations may not be appropriate for all participants, and that good communication between researchers and participants is essential for reducing biases in participation and data acquisition in neuroscience research.

Below we discuss considerations surrounding hair, but such discussions are not replacements for lived experience^20,21^. It is essential that labs employ research staff, both for participant-facing data collection, as well as for data analysis and study design, who identify similarly to the demographic of the participant population. This has the benefit of increasing the cultural appropriateness of protocols, participant experiences, and interpretation and communication of findings. When this is not possible, community members and leaders should review study materials and protocols.

## Preparing families for a pediatric EEG recording

The goal of any research team working with a diverse sample of participants should be to collect generalizable, representative, and high-quality data. To ensure this, families should feel like best practices are being used and laboratories should spend time developing materials and messaging that helps all families feel informed, autonomous, and well-prepared for their EEG visit. It is essential that laboratories use language and dialects that are familiar to participants – this is particularly salient for participants who identify as Latinx, as different regional dialects of Spanish can use different wording for similar concepts. Conversations about EEG, its purpose, and how hair can and should be styled need to be informed, sensitive, and begin well before the lab visit, so that families enter the lab with some level of understanding. There are varying ways a lab can distribute information to families, and researchers should assess which method(s) will work best for their participants. Here, we suggest best practices for reducing the disproportional exclusion of Black and Latinx participants in pediatric EEG data collection through various channels of communication used to discuss EEG and hairstyling with participants prior to a lab visit. Additionally, we provide example materials created by our research team for others to use.

Over the past five years, a growing number of resources and webinars (e.g., Richardson & Black in Neuro, 2021) have emerged focusing on hair and EEG research in adulthood from groups such as Black in Neuro (blackinneuro.com), SPARK Society (sparksociety.org), and the Hello Brain Lab (https://hellobrainlab.com). There has also been one webinar focused on older children by Dr. Hudac and Magstim/EGI^22^. Here we build on these recommendations with a focus on conducting EEG visits with infants and very young children and their families, which commonly requires more planning (e.g., children are less receptive to in-lab styling) and strong lines of communication with both parents and children.

For most families, an EEG recording is a novel experience. Like many medical and scientific procedures, the purpose and equipment involved in the assessment can feel invasive or difficult to understand, leading to potential parent apprehension – particularly for parents belonging to racial or ethnic groups that have experienced historic injustices and racism in scientific research^21,23^. Families may be concerned about what EEG measures and why it is collected. Furthermore, once families learn that the EEG cap must fit closely to the head, the EEG procedure involves adding salt water or gel to the hair, and that the EEG process will likely disturb or ruin existing hairstyles, potential participants are more likely to decline EEG participation. Furthermore, participants may also worry that the lab is not well prepared to approach their hair. This is a particular risk when participants and the researchers are not matched according to race and/or culture.

To successfully collect pediatric EEG, it is essential that researchers, parents, and participants have a shared understanding of the goals of EEG cap application. In particular, there is a delicate balance that needs to be achieved between getting optimal scalp and hair conditions for EEG recording and participant ease and comfort. These two priorities are more likely to be at odds with families who identify as Black and/or Latinx due to both equipment limitations (see section entitled *Equipment Considerations* for further discussion) and hair practices/styles that impede the EEG signal. Briefly, examples of specific hair considerations include the use of heavy hair oils, conditioners, and products that may increase recording impedance and voluminous or raised hairstyles that will prevent the cap from easily contacting the scalp. In the following sections we share what we have learned as well as resources we developed that increase the likelihood of parent consent, child assent, and high-quality data collection.

### Hair considerations before a research lab visit

When bringing participants into an EEG research lab, it is common to request that participants’ hair be clean (e.g., free of styling product) and worn down (or in a style that can be easily taken down). These two seemingly simple requests can pose unanticipated burdens for Black and Latinx participants.

#### Conversations around cleanliness

Conversations around hair cleanliness can be particularly sensitive and difficult. First, asking participants to come to the lab with “clean” hair may inadvertently validate the harmful stereotype that Black/Latinx hair may be unclean to begin with. There is a long-standing racist history surrounding Black hair, as well as contemporary reports of hair-related bullying as well as micro- and macroaggressions, particularly in educational settings^24–26^. While the genesis of bullying, microaggressions, and macroaggressions vary, many narratives surround kinky, coily, or dense wavy hair types being unclean and/or unkempt particularly when worn in its natural state (e.g., without protective styles or the use of heavy styling products). As such, we recommend against references to cleanliness in participant communications.

Second, by asking Black or Latinx participants to come to the lab with “clean” hair, researchers are, perhaps inadvertently, asking families to wash their child’s hair before their visit. Hair washing can be a time-consuming and sometimes stressful process, which places a burden on the participant and their family. Additionally, for most EEG recordings, families will likely need to wash their child’s hair twice – once before the recording to have “clean” hair, and once after the recording to remove conductive–media (e.g., saline or gel). While washing a child’s hair twice in close proximity may not be too burdensome for participants with fast-drying hair or close-cut hairstyles, this request may be particularly burdensome for Black and Latinx participants, for whom the washing, detangling, and styling process can take hours, be uncomfortable or painful, and can cause hair or scalp dryness.

#### Conversations around hair style

Asking participants to come to the lab with their hair worn down poses three major problems.

First, the natural hair of child participants who identify as Black and/or Latinx is, on average, more voluminous, curly, and may less likely to absorb liquid than White hair (for an excellent review on Black hair see Choy et al.). As such, in comparison with participants who identify as White, participants who identify as Black and Latinx are more likely to spend more styling their hair these styles may be practical as well as a means of self-expression. It is not uncommon for child participants to not feel comfortable having their hair “down” as it may fall in their face, stick to their neck, not stay behind their ears, or feel itchy. Furthermore, asking participants to wear their hair “down” may make participants feel uncomfortable or unkempt. This is particularly true given that there can be stigma around having hair that appears “frizzy” or “messy” in Black and Latinx communities. As a result, some families may decide to straighten or similarly style their child’s hair, to present it as “clean” and “down”, but even straightened styles can be time consuming and hold a lot of value for participants.

Second, researchers may unknowingly be asking participants to remove permanent or long-lasting hairstyles which may not be possible or ready to be removed. Black and Latinx participants are more likely to wear long-lasting, protective, and/or permanent styles, which makes requests for removal or disruption by the EEG procedure an impediment. As an example, in some Latinx communities (in particular, Black Caribbean-Latinx communities) children style their hair in intricate styles that include braids and/or twists that can last weeks and should be washed sparingly. Similarly, permanent (e.g., locs) and long-lasting (e.g., cornrows, twists, braids) hairstyles are common in Black communities even in early childhood. The process of washing and styling hair can be incredibly time-consuming (often hours, including detangling long hair) and expensive (e.g., child scalp braid styles can range from $50–$200 in urban salons). These hairstyles are commonly intended to last weeks or months. By asking participants to arrive to the lab with their hair “down,” researchers are asking families to remove these styles, which leaves many parents with the perception that they need to either remove their child’s hairstyle or refuse EEG collection. In the case of more permanent styles like locs, removal is not possible, and thus participants are likely to decline EEG collection. It is essential that participants are offered options around how to style their hair that will make them feel confident as well as work well for EEG collection.

Third, in addition to day-to-day styles, a vital discovery for our team was the concept of “birthday hair” or “event-related hairstyles.” In pediatric and developmental research, it is common to time data collection around a child’s birthday (e.g., 3-year data collection when a child turns 3). The coincidence of a child’s birthday and data collection can be problematic, as we discovered that children who identified as Black and/or Latinx would sometimes get special, longer-lasting birthday hairstyles (e.g., a special twist, braid, or blowout for their birthday celebration). These birthday hairstyles were more likely to be styled or installed by a professional and cost more than a child’s style during other times of the year. In addition to birthday-specific styles, other annual calendar events were also more likely to prompt these special styles, including Thanksgiving, Christmas, graduation, and the first day of school. Families are much more likely to refuse EEG recordings if they would ruin a child’s birthday or event-related hair. It is essential to have procedures in place to communicate with families before their visit to the lab about current/intended hairstyles and to have flexibility surrounding when families come into the research lab.

### Conversations during scheduling

Talking to families over the phone is often the most successful method of communication between the research team and participants. However, if not handled competently, phone conversations can immediately signal to participants that the research team is not equipped to deal with different hair types. In our experience, it is helpful for lab staff to begin the conversation with a brief overview of EEG before discussing hairstyling. It is important to name the method, explain its purpose, and explain what the EEG equipment will and will not do (e.g., “*EEG does not send anything harmful into or change your child’s brain”* and “*the little sensors or electrode just ‘listen’ like little microphones”*). This overview should be brief, in order to value the listener’s time, but also to reduce their cognitive load given that all the information may be brand new. However, a key piece of information that should be conveyed is that, to get a valid reading, EEG caps need to sit close to the scalp, like a winter hat or swim cap, which can be made easier by styling their child’s hair in certain ways.

After a brief overview of EEG, it is usually a good time to ask families if they are comfortable providing more information about how they typically style their child’s hair. To have this conversation, it is essential that the research team be familiar with common types of participant hairstyles, and be ready to engage in the conversation in a non-judgmental and competent manner. While one may be tempted to have an open, unstructured conversation, such conversations can easily end in miscommunications or differences in terminology, resulting in reduced participant confidence in the research team. We suggest creating a brief, standardized calling script that is culturally safe, then piloted and reviewed by members of the participant community. Supplements 1 and 2 show example scripts used by the Baby’s First Years project^27^, which were reviewed by researchers, consultants, members of the community, and pilot participants drawn from the intended participant population.

**Fig. 1:**
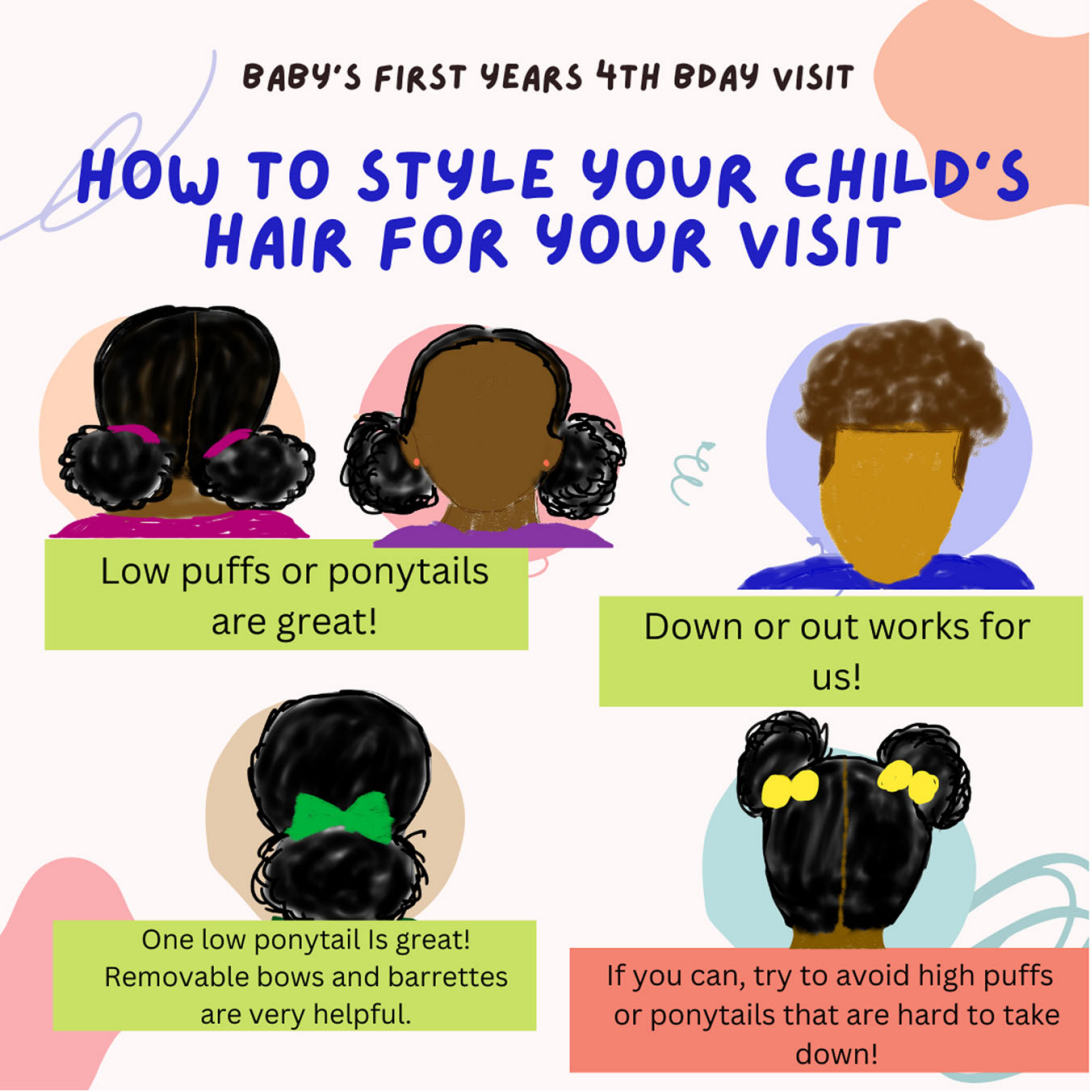
Examples of how to style a child’s hair for their EEG visit.

The goal of scripts for telephone calls prior to lab visits should be fivefold. First, these scripts should tell parents about EEG. Second, they should get information about how a child typically wears their hair. Third, if a child typically wears their hair in a long-lasting style (e.g., braids) that is meant to be removed only every so often, researchers should offer to schedule their visit around the next time the child’s hair is about to be styled. Alternatively, if the style will not be removed or the child has a more permanent style (e.g., locs), researchers should discuss whether the parents would be comfortable capping over the style (implications for signal quality discussed later in this article). Fourth, if the child’s hairstyle is likely to impede the cap from making good contact with the scalp (e.g., puffs or natural hairstyles), the researcher should provide suggestions and examples (see Fig. 1) of styles that are optimal for capping. Finally, the family should be informed that the child’s hair will need to be washed after the visit, due to the conductive media of the EEG cap (e.g., gel or saline). This is essential information, particularly for Black and Latinx participants, given that the washing and styling process can be time-consuming.

If executed well, the pre-lab visit hair conversation will make it more likely that Black and Latinx families will schedule their visit at a time that is conducive to EEG recording (e.g., when styles are removed or near a planned wash day). It also ensures that families aren’t asked to remove or ruin special hairstyles on the day of the visit or wash their child’s hair unexpectedly. In addition, it provides research staff with valuable information surrounding how families are feeling about EEG and the equipment/supplies that may be needed.

### Providing a video explaining EEG

Some families will prefer to see what EEG capping and collection looks like before their visit and/or before they consent. Providing a brief video gives families a better sense of what the EEG process looks like, as well as the space and overall atmosphere (See Supplements 3 and 4). Labs should focus on creating or using a video that strikes a balance between using simple language and providing useful details. Especially for studies with child participants, the video should be fun and appealing. Having families approach the EEG process with an understanding that it is safe to ask questions and an idea of what is about to happen can make the whole process more enjoyable for all. One of the most powerful tools for reducing disproportional exclusion of Black and Latinx participants is being able to show families real examples of successful visits with participants from similar racial or ethnic backgrounds. Including short clips of a variety of families, with different hair types and styles, will assure participants that the lab is well-equipped to approach their hair with comfort and care. The lab may choose to send participants the video before a call (providing a chance to ask questions about the video) or after a call (to give a visual of some of the concepts discussed on the phone) depending on what the family may prefer.

### Valuing feedback

Given that lab staff will know more about the measure and process than the family, it may be tempting to approach communication almost as a lecture – providing a good deal of information with little response from the participant. However, to reduce race-related biases in terms of who consents to and completes EEG data collection, it is important to value the feedback and thoughts of the families who know the most about their own values, feelings, and apprehensions. Lab staff should be continually open to refining and renewing their communications with help from the families themselves. For example, a group of participants might express that they are uncomfortable undoing a specific hairstyle the night before the assessment. Staff can use this feedback to have useful conversations with future families (*“We’ve heard from other parents that ______ hairstyle can be challenging to remove the night before. Would you like to schedule around a time when you’re restyling your child’s hair?”*) Staff should ensure families that they are in control of the process and are valued partners in the experience.

## Conducting a pediatric EEG lab visit

### Equipment considerations

When considering how to accommodate a diversity of hair types, researchers face two options – either adapt their EEG system to accommodate different hair types, or adapt participant hair to the accommodate the EEG system. Historically, most pediatric EEG research has attempted to do the latter, by encouraging participants to style their hair in different ways. However, many of the racial and ethnic biases in EEG research stem from the design of the EEG hardware, with most issues coming from the form factor of caps and electrodes.

**Fig. 2:**
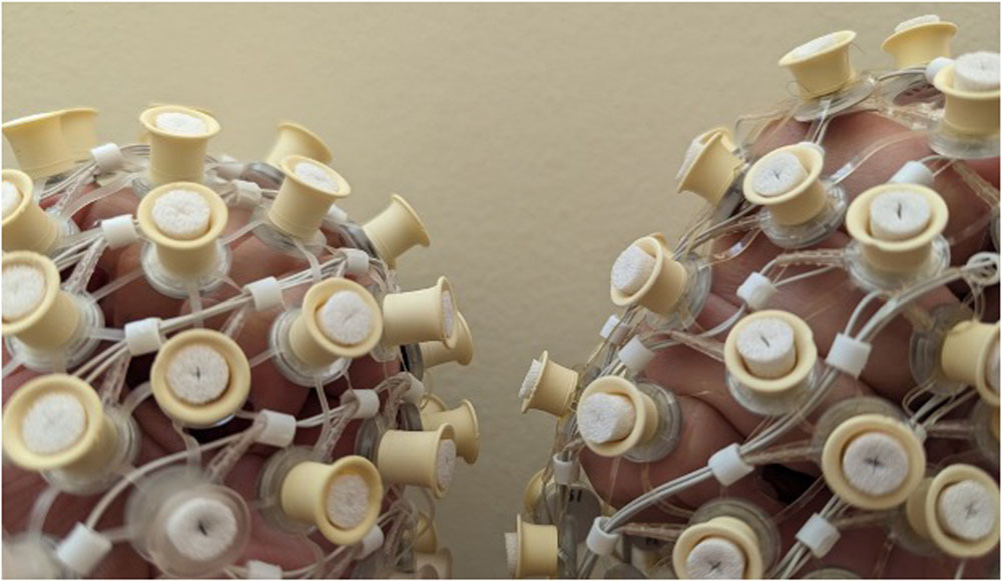
EEG Cap modified to work with dense, curly hair types by elongating sponges and pedestals (left) compared to typical EEG cap with shorter sponges and pedestals (right).

Most of the widely available electrode types and caps for pediatric EEG are not well-suited for data collection with dense, curly, or thick hair^17^. The primary limitation for most caps and electrodes available on the market is that the allowed space between the electrode and scalp is not wide enough to accommodate thick hair or raised hairstyles like braids. Specifically, many systems only allow a centimeter or two of space between the electrode and scalp to accommodate hair (see Fig. 2). On some systems, this limited space can be expanded slightly by pulling hair through cap holes or cap webbing. However, pulling hair through the cap can be time consuming, and doing so is not always tolerated by young children (this is particularly true if the process takes time, requires tugging, or if the child has negative associations with hairstyling).

Some promising, steps are being taken to reduce systemic racism in EEG electrodes. For example, Etienne and colleagues designed a novel electrode for reliable EEG recordings on coarse and curly hair (Etienne et al.). However, these electrodes are not currently available for most pediatric EEG systems, and require a specific braiding pattern, which can be time consuming and difficult for children. In addition to novel electrodes, some companies are beginning to allow cap customizations for dense, coarse, and curly hair. For example, Magstim EGI (Magstim EGI, Eugene, OR, USA) now allows for ordering caps with elongated pedestals, which allow for more space for hair between the cap and scalp (Fig. 2). Additionally, dry comb electrodes are beginning to enter the market. Besides the distinct advantage of being dry and not requiring post-visit washing, the stiffness of these comb electrodes and the small footprint at the end of each comb tine allows for easier penetration through the hair, and even the ability to penetrate long-lasting and permanent styles like braids and locs. Finally, exciting new efforts are underway to account for individual differences in hair texture and volume analytically^28^.

### Considerations for capping different hair textures and styles

The ease of capping will certainly differ by hairstyle, texture of the child’s hair, and the child’s comfort with research staff touching their head (for excellent resources on adult capping see Etienne et al., Richardson et al., and Richardson & Black in Neuro).

It is important to consider the psychological framework that a child may have when being approached with an EEG cap. For some Black and Latinx children, hair styling can be a negative experience. Many young children may have sensitive scalps (colloquially referred to as being “tender-headed”). The combination of a sensitive scalp and thick, coarse, of voluminous hair can make washing and detangling uncomfortable or even painful for children. Because of this, many children have built up hesitation or fear around any hair-related activity and may get upset when their head is approached. In instances where prior negativity around hairstyling makes a child feel hesitant or fearful, it is essential to talk openly and honestly about the cap, let the child explore the cap if that seems helpful, and communicate clearly that they can stop the capping process at any time. If researchers look for and acknowledge a child’s hesitation around having their hair and head touched and give them reassurance and autonomy in the capping process, they are much more likely to reduce race-related differences in EEG capping rates and data collection.

In addition understanding a child’s reaction to being approached with a cap, it is also important to understand that asking families to restyle their child’s hair before capping can be sensitive. The act of hair-combing and styling can be very intimate, with physical closeness, touching, and patting, as well as a time of racial socialization^29,30^. As such, parent’s should always be provided with the opportunity to restyle hair before lab staff attempt any hair style alterations.

Overall, lab staff will want to try to find the method for collecting the best data while also avoiding too much adjustment and interference with the child’s hair. While by no means exhaustive, here we discuss considerations around a few styles that are frequently seen in young children, including scalp braids, individual plaits or twists, loose natural styles, puffs, ponytails, locs, and blow outs.

#### Scalp braid styles

Styles in which hair is braided to the scalp can be very convenient for parents and children, because they typically require little to no daily prep and can last for weeks at a time. However, these styles can also be challenging for EEG cap application and obtaining high-quality recordings, because the hair is typically not moveable and parents may not be able to reschedule around the style (e.g., it could be over a month until the style is taken down, or they may have a very short transition time between styles). If a parent informs the staff that their child’s hair may be braided to the scalp during the visit, staff may begin by asking if there might be a better time to complete the recording. It could be the case that the parent planned on taking the style down soon and has no issues with scheduling with that in mind. However, this could be difficult for both the family and lab staff to work around, as plans and schedules can often change unexpectedly. It is important to note that staff should not ask parents to remove scalp braid styles during the visit unless previously discussed. Asking families to remove these long-lasting hairstyles can place parents in an uncomfortable position, as they may have to weigh pleasing the research team against the time, value, and possible child discomfort that comes with removing scalp braid styles. If scalp braid styles are not scheduled around or removed, the lab staff may try proceeding with the recording by capping over the scalp braids.

In the event the research team, parent, and child agree to cap over a scalp braid style, it is important to reiterate that the child’s hair should be rinsed after the visit if the child feels itchy, similar to how they might feel after swimming in the ocean or a chlorinated pool. Once the cap is applied, researchers may notice that scalp braid styles may be helpful, because areas of the scalp are exposed between the braids. In these cases, researchers should aim to capitalize on open areas of scalp by maneuvering electrodes over these open areas to reduce impedance. However, there will be other areas where an electrode may sit on top of hair that is immovable, prohibiting the electrode from contacting the scalp. When researchers are met with electrodes sitting on top of braids, it is essential that researchers are careful not to forcefully push the electrode into or around the braid, as that can be painful for the participant. Ultimately, for scalp braid styles, it is our recommendation that the research team focus on making contact with the scalp where possible, and noting for data analysts which electrodes that have less optimal contact.

#### Individual plaits, braids, or twists

Another popular style for young Black and Latinx children may be styles in which small sections of hair are braided or twisted and typically hang from the scalp. These styles can range widely in the number of individual braids or twists, the length, and added accessories. However, these styles are typically easier for EEG recordings than scalp braids because the individual braids or twists can be lifted and gently pulled through the cap. This style may also expose some of the scalp, possibly under the twist or braid itself, depending on how recently the style was installed (less recent installations result in easier scalp access). Finally, as with scalp braid styles, parents should never be asked to remove these styles at the visit without warning, as removal can be time consuming, painful, and stressful for the participant.

It is also common for parents to add accessories to these styles (beads, barrettes, balls). Some accessories are easy to remove, and may even be replaced before the family leaves the lab. Other accessories (e.g., stacked beads) can take a good deal of time to install, and will not be as easy to replace. The key to navigating these accessories is to begin the conversation with parents early, and to not surprise parents and participants with last-minute requests to remove accessories. This means, in pre-visit communications, research staff should inquire whether the child typically wears accessories as a part of their braided styles and, if so, what those accessories usually are. If the accessories are items like balls or barrettes, ask the parents if they would be comfortable either removing them at home or during the visit. This provides parents with time to consider what would be easiest for them and their child.

#### Loose and natural styles

Many families will opt for their child’s hair to be “down” or “out” either as a daily style, or as a deliberate choice to take down another hairstyle before their visit. These styles are the most flexible for EEG data collection, typically allowing best access to the scalp, as well as allowing for adjustments to be made with the EEG cap.

For longer styles, and if texture allows, the lab staff may ask the parents if tying hair into two or more low ponytails prior to applying the cap is acceptable. These low ponytails allow for easier access for applying the cap by keeping hair flush to the head and allowing the experimenter to see fiducials while also giving a better chance of hair not getting into the child’s face as the cap is being applied and adjusted. If the child’s hair length, texture, or tolerance for adjustment prevents the use of ponytails, staff can proceed with placing the cap directly over the child’s hair. In these cases, it may be helpful for additional research staff to help compress hair or stretch the cap over more voluminous styles. Ensuring the cap is situated comfortably but also snugly on the head and pressing the electrodes to the scalp is essential for high-quality data collection. To improve scalp contact for the electrodes, a soft pipette or similar tool can be used to help move hair out of the way.

#### Puffs or ponytails

If parents inform the lab staff ahead of time that their child typically wears their hair in puffs or ponytails, this provides a great opportunity to ask if they can adjust the puffs or ponytails to be as low as possible on the head (i.e., toward the nape of the neck). Puffs or ponytails that sit directly on top of the head can pose the greatest challenge, as they are commonly too large to pull through the cap and too raised off the scalp to enable electrode contact. Additionally, the removal of higher puffs or ponytails can be quite stressful to child participants –especially if the child is resistant to having their hair taken down and adjusted. In these cases, lab staff should ask parents the most optimal time for their child’s hair to be adjusted (i.e., at home or in the lab) to ensure the best chance of collection.

#### Locs

Loc styles can be some of the most advantageous styles for families, as they may require less daily maintenance than longer-lasting braids or temporary styles. In these cases, parents should not be asked if they are willing to remove the style or reschedule for another time, as there is typically no participant intention to remove loc styles. Depending on the length and type, some locs may be able to be tied into low ponytails, much like loose hair. Access to the scalp may also depend on the style and how recently locs were retwisted or interlocked. In these cases, it is important to work with families to establish a level of comfort around moving individual locs as well as washing or rinsing the locs after the visit. Like hanging braids, it can be possible to pull locs through cap holes or webbing to get the cap to sit flush with the scalp.

#### Slicked back, relaxed, blown out, or straightened hairstyles

Blowouts and/or straightened hairstyles are very common, particularly among families who identify as Latinx (although similar styles are not uncommon among participants who identify as Black). The phenotype of “Latinx hair” is vast, with hair textures ranging from smoother, straighter hair to denser, curlier hair. Historically, long, smooth, straight or relaxed, and shiny hair has been a beauty standard among many cultural groups identifying as Hispanic or Latinx^31^. As such, it is common for participants who identify as Latinx to have hairstyles that are slicked back, relaxed, blown out, or straightened. For participants with thicker and curlier hair, these styles can take a long time to achieve (e.g., it is not uncommon for straightening through the use of blow drying, hair rollers or both to take well over an hour) and may be costly (e.g., professional blowouts can easily cost $50 or more for long hair). Given the time and money that can be involved with achieving these hairstyles, they are sometimes meant to last several days to a week or more. Furthermore, these styles may be painful for children to endure, as the high heat settings commonly used for blowouts and hair dryers, and the chemical relaxants may irritate or burn the scalp. Similarly, achieving slicked-back hairstyles often requires firm brushing and the tight tying of hair, which can be painful and lead to increased scalp sensitivity. This increased sensitivity should be considered when performing procedures like pulling the cap across the scalp, moving hair with pipettes or combs, and/or abrading the scalp. It is important to consider that even simple hairstyles (e.g., worn down) may hold a lot of value to participants, and asking participants to get their child’s hair wet or to ruin their hairstyle may be met with hesitation or refusal. While placing the cap with these styles is usually rather simple, it is important for interviewers to understand whether participants are ready for the style to be disturbed or ruined and to be gentle with the child’s scalp.

#### Research team flexibility with different hairstyles

It is key for lab staff to keep in mind that hairstyles, texture, and length can vary widely amongst different children. What works for one child may not be successful with another. For example, some children may feel comfortable with research staff touching their hair, while others may be more comfortable with their parents handling their hair. Staff should be familiar with how to remove common hair accessories, gently tie hair into ponytails, and manipulate and part a variety of hair textures. If staff encounter a moment where they are not sure how to handle a participant’s hair, it is essential they create a culturally safe and open conversation with children and families to come to the best solution to navigate getting the cap close to the scalp without discomfort and burden. Ultimately, to increase inclusion in pediatric EEG research, parents, child participants, and the research team must begin conversations surrounding hair before the visit even begins. Communication, flexibility, and openness to feedback must continue throughout the in-person EEG protocol.

### Ending the EEG visit

Many research teams may assume that the end of the EEG recording is when steps to reduce racial and ethnic biases in pediatric EEG may cease. However, the end of data collection does not mean the experience of the EEG is over for the family; the participant’s continued wants and needs must be considered, both to increase the likelihood of future research participation, and more generally to improve relations between scientists and Black and Latinx participants. In particular, we find two things need to be addressed with families before they leave the lab. First, after the EEG cap is removed, the child’s hair is likely to be disheveled. While this may not be stressful for some participants, others may feel uncomfortable leaving the lab with wet or unstyled hair. We recommend creating a “hair bar” for participants, including disposable combs, hair dryers, and hair products that are familiar to the populations from which participants are drawn. Such products vary regionally and by cultural identity. Examples of products may include small rubber bands for styling, hair ties, parting combs, brushes designed to reduce pull or hair breakage, hard-casting gels, curl creams, a diffuser for the blow dryer, a flattening iron, a round brush, and edge stylers. We encourage research teams to conduct focus groups or ask pilot participants about the products and tools that would be most helpful, as well as the places in their community where such products are commonly purchased. Second, we recommend asking participants at the end of the visit if they have any lingering questions about the data collected and next steps. Participants commonly have new questions about the brain data, tasks, or data handling after seeing the EEG data collection. It is essential that participants leave the lab with a clear understanding of why the data were collected, where the data are going next, and what can be learned from their child’s participation.

## Recommendations for future pediatric EEG research

There is still much work to be done beyond the recommendations made here to reduce racial and ethnic biases and unintentional systemic racism that plague pediatric EEG research which, in turn, produces less generalizable and representative data used for neuroscience-backed education and policy work. While discussions detailing racial and ethnic biases in EEG research are growing (e.g. Bradford et al., Choy et al., and Etienne et al.), it is difficult to directly measure these biases. It is not uncommon for participant demographics to be omitted from papers, and participant attrition from neuroscientific measures is seldomly reported by race^32^. Additionally, while not covered in the current review, there are likely differences in participant exclusion by race in post-collection data processing of EEG data. One place this is particularly apparent is in epoch rejection, as hairstyles such as scalp braids make it much more likely to exceed interpolation thresholds, thereby rendering it likely for participant data to be excluded from analysis based on race. Third, it is unclear how specialized electrodes, increased pedestal lengths, increased scalp oils from not pre-washing hair, and other modifications made to increase inclusion in EEG data collection may impact the EEG signal. Basic methodological work should be conducted to establish the validity of these techniques intended to reduce race-related bias and systemic racism in pediatric EEG.

## Conclusions

Increasing diversity and inclusion in pediatric EEG data collection is paramount for reducing systemic racism in neuroscience data used to understand educational inequalities and inform policy. To address the inequities in the pediatric EEG literature, scientists should prioritize diversity and inclusion through their recruitment practices, staff selection and training, research materials, equipment choices, lab supplies, and visit protocols. While such efforts may be underappreciated by the broader scientific community, they are essential for the most robust and generalizable neuroscientific data needed in intervention research and policy formulation.

### Reporting summary

Further information on research design is available in the Nature Research Reporting Summary linked to this article.

### Supplementary information

Supplemental Materials

Reporting summary
