# Supplementary material for: Fostering inclusion in EEG measures of pediatric brain activity
Source: NPJ Sci Learn. 2024 Apr 2;9:27. doi: 10.1038/s41539-024-00240-y (PMC10987610; doi:10.1038/s41539-024-00240-y)
Supplement: Supplementary file 1 — Supplemental Materials [file 41539_2024_240_MOESM1_ESM.pdf]

Supplementary Notes:

Supplement 1: *Longer EEG Hair Script for Phone*

English

1) **Experimenter:** During your visit to the lab, we would like to collect a measure of brain activity called EEG to look at how your child's brain is growing. To look at your child's brain activity, we would place a soft, stretchy cap specially designed for children on their head. The cap that we will use is very safe for your child to wear and does not send anything into your child's brain. You don't need to decide right now whether you would like to complete the EEG recording, and we will give you much more information about the EEG when your visit the lab.

   However, if you think you might like to participate in EEG, I would like to ask you a few questions about how you usually style your child's hair in order to understand how the EEG cap might fit. Specifically, we would like the cap to fit as close to the head, like a swim cap or tight winter cap. Is that okay with you?

   a) **Mom Response of 'Yes':** Great!

      My first question is whether you currently or usually style your child's hair in a hairstyle that lasts for multiple days or weeks at a time? Like braids or locs. Or does your child usually wear styles that would prevent the cap from sitting against the head? This would include styles that have accessories like bead or large ties.

      i) **Mom Response of 'Yes':** Good to know! When we put on the EEG cap, it is easiest if your child's hair isn't in one of these styles because the cap cannot sit tightly against their head. So, if possible, we would like to schedule your visit at a time right before you restyle your child's hair. We understand this isn't possible for all styles, do you think that would be possible for your child?

         (1) **Mom Response of 'Yes':** Great! When thinking about your visit, it would be easiest if you were able to remove your child's hairstyle before coming to the visit. Do you think that would be possible?

            (a) **Mom Response of 'Yes':** Perfect. Additionally, the EEG cap is soaked in a mild saltwater with a little bit of baby shampoo. The saltwater isn't very salty – about as salty as tears. Because of the salt water, some children experience an itchy head after the water dries, like what you might experience after swimming in a chlorinated pool or going to the beach. As such, we recommend washing your child's hair within a day or two after the visit. Given your child's current schedule, do you think that would be possible?

               (i) **Mom Response of 'Yes':** Great! When we call a day or two before to confirm your visit, we will be sure to remind you not to style your child's hair in any special way and have them wear an easy style (like down or in a ponytail) with no product. ***[Continue to schedule visit around when child's hair will be ready to be restyled].***

(ii) **<u>Mom Response of 'No':</u>** Okay, no worries. On the day of your visit, we can show you the cap, give you more information, and decide whether it makes sense to try on the EEG cap.

(b) **<u>Mom Response of 'No':</u>** Okay, no worries. On the day of your visit, we can show you the cap, give you more information, and decide together whether it makes sense to try on the EEG cap.

(2) **<u>Mom Response of 'No':</u>** Okay, no worries. On the day of your visit, we can show you the cap and decide together whether it makes sense to try on the EEG cap.

ii) **<u>Mom Response of 'No':</u>** Okay, great then the cap should fit over their hair. Additionally, the EEG cap is soaked in a mild saltwater with a little bit of baby shampoo. The saltwater isn't very salty – about as salty as tears. Because of the salt water, some children experience an itchy head after the water dries, like what you might experience after swimming in a chlorinated pool or going to the beach. As such, we recommend washing your child's hair within a day or two after the visit. Given your child's current schedule, do you think that would be possible?

(i) **<u>Mom Response of 'Yes':</u>** Great! When we call a day or two before to confirm your visit, we will be sure to remind you not to style your child's hair in any special way and just to have them wash their hair and wear it in a style that is easy to take out (like down or in a ponytail).

(ii) **<u>Mom Response of 'No':</u>** Okay, no worries. On the day of your visit, we can show you the cap, give you more information, and decide together whether it makes sense to try on the EEG cap.

b) **<u>Mom Response of 'No' or 'Not interested in EEG':</u>** Okay! No worries. When you come into the lab, we can give you more information about EEG and we can decide together whether putting on the EEG cap makes sense.

Spanish

1) **<u>Experimentador:</u>** Durante su visita al laboratorio, nos gustaría recolectar la actividad cerebral (EEG) de su hijo/a para observar cómo se está desarrollando su cerebro. Para medir esta actividad cerebral, colocaremos en la cabeza de su hijo/a un gorro suave y elástico diseñado especialmente para niños. El gorro que usaremos es muy seguro y no envía nada al cerebro de su hijo/a.

   No es necesario que decida si desea participar en la grabación del EEG en este momento. Le brindaremos más información sobre esta actividad cuando visite el laboratorio. Sin embargo, si usted cree que le gustaría participar en el EEG, me gustaría hacerle algunas preguntas sobre cómo suele peinar el cabello de su hijo/a para entender mejor cómo le quedaría el gorro de EEG. Idealmente, nos gustaría que el gorro se ajustara lo más cerca posible a la cabeza, como un gorro para nadar o un gorro ajustado de invierno. ¿Está usted de acuerdo?

a) **<u>Si la mamá responde 'Si':</u>** ¡Excelente!

Mi primera pregunta es ¿actualmente o habitualmente peina a su hijo/a con un peinado que dure varios días o semanas? Como trenzas o rastas. ¿O su hijo/a suele usar peinados

que impidan que el gorro se ajuste a su cabeza? Esto incluiría peinados que tengan accesorios como cuentas o gomitas grandes para el cabello.

i) **<u>Si la mamá responde 'Si':</u>** ¡Gracias por dejarnos saber! Cuando le pongamos el gorro de EEG a su hijo/a, es más fácil que el cabello de el/ella no tenga uno de estos peinados ya que esto va a impedir que el gorro quede bien ajustado en la cabeza de su hijo/a. Por ese motivo, si es posible, nos gustaría programar su visita justo antes de que usted vuelva a peinar a su hijo/a. Entendemos que esto no es posible para todos los peinados, ¿crees que sería posible para su hijo/a?

(1) **<u>Si la mamá responde 'Si':</u>** ¡Excelente! Pensando en su visita, lo más fácil sería si pudiera quitarle el peinado a su hijo/a antes de venir a la visita. ¿Crees que eso sería posible?

(a) **<u>Si la mamá responde 'Si':</u>** Perfecto. Además, el gorro de EEG se moja en agua un poco salada con un poco de champú para bebés. El agua no es muy salada, casi igual de salada que las lágrimas. Debido al agua salada, algunos niños sienten picazón en la cabeza después de que el agua se seca, como la que se podría sentir después de nadar en una piscina con cloro o ir a la playa. Por lo tanto, recomendamos que le lave el cabello a su hijo/a uno o dos días después de la visita. Teniendo en cuenta la rutina de su hijo/a, ¿cree que eso será posible?

(i) **<u>Si la mamá responde 'Si':</u>** ¡Excelente! Cuando llamemos uno o dos días antes para confirmar su visita, nos aseguraremos de recordarle que no le peine el cabello a su hijo/a de ninguna manera especial y que use un peinado fácil (por ejemplo suelto o en una cola) sin producto.***[Continue to schedule visit around when child's hair will be ready to be restyled]*.**

(ii)**<u>Si la mamá responde 'No':</u>** Bien, no hay problema. El día de su visita, podemos mostrarle el gorro, brindarle más información y decidir si tiene sentido probar el gorro de EEG.

(b) **<u>Si la mamá responde 'No':</u>** Bien, no hay problema. El día de su visita, podemos mostrarle el gorro, brindarle más información y decidir si tiene sentido probar el gorro de EEG.

(2) **<u>Si la mamá responde 'No':</u>** Bien, no hay problema. El día de su visita podemos mostrarle el gorro y decidir juntos si tiene sentido probar el gorro del EEG.

ii) **<u>Si la mamá responde 'No':</u>** Perfecto, entonces el gorro debe quedar sobre su cabello. También, el gorro del EEG se moja en agua un poco salada con un poco de champú para bebés. El agua no es muy salada, casi igual de salada que las lágrimas. Debido al agua salada, algunos niños sienten picazón en la cabeza después de que el agua se seque, como la que se podría sentir después de nadar en una piscina con cloro o ir a la playa. Por lo tanto, recomendamos lavarle el cabello a su hijo/a uno o dos días después de la visita. Teniendo en cuenta la rutina de su hijo/a, ¿cree que eso sería posible?

(i) **<u>Si la mamá responde 'Si':</u>** ¡Excelente! Cuando llamemos uno o dos días antes para confirmar su visita, nos aseguraremos de recordarle que

no debe peinar el cabello de su hijo/a de ninguna manera especial y solo pedirle que le lave el cabello y use un peinado que sea fácil de quitar (por ejemplo suelto o en una cola).

(ii) **Si la mamá responde 'No':** Bien, no hay problema. El día de su visita podremos mostrarle el gorro, brindarle más información y decidir juntos si tiene sentido probar el gorro de EEG.

b) **Si la mamá responde 'No' o 'No estoy interesada en el EEG':** ¡No hay problema! Cuando venga al laboratorio, podemos brindarle más información sobre el EEG y podemos decidir juntos si tiene sentido probar el gorro de EEG.

Supplement 2: Brief English Hair Script for Phone

English

1. **Experimenter:** We would like to collect a measure of brain activity called EEG to look at how your child's brain is growing. To look at your child's brain activity, we would place a soft, stretchy cap specially designed for children on their head. The cap that we will use is safe for your child to wear and does not send anything into your child's brain. You don't need to decide right now whether you would like to complete the EEG recording, and we will give you more information about the EEG when you visit the lab. However, if you think you might like to participate in EEG, I would like to ask you about how you usually style your child's hair in order to understand how the EEG cap might fit. Is that okay with you?
    a. **Mom Response of 'Yes'**: How does your child usually wears their hair or how they may have it styled when you visit? (RA note: If examples are needed, you can say: Which of the following best describes your child's usual style?
        1. Close-cut (buzz-cut, fade, waves, Caesar-cut, etc.)
        2. Down or out
        3. Ponytails, buns, or puffs
            a. IF Ponytails, buns, or puffs: Discuss whether mom can avoid use of accessories or remove them on the day of the visit. Also consider sending styling suggestions.
        4. Braids (this could be their own hair or feed-in)
            a. IF BRAIDS: Good to know! If you'd like, we can try to schedule around a time when their hair is out or down. Or, we can proceed with the visit with their hair in that style. What do you think?
        5. Locs
            a. IF LOCS: Good to know! We can make sure to try to work around their locs to get the best measure or we can talk about it more on the day of your visit. What do you think?
        6. Other
            a. How do you think a cap (like a swim or shower cap) would fit against their head with this style?

    ii. **Mom Response of 'Yes'**: Is there anything else you would like us to know about your child's hair?

iii. **<u>If mom still seems interested:</u>** Perfect. Additionally, the EEG cap is soaked in mild salt water with a little bit of baby shampoo. The saltwater isn't very salty - about as salty as tears. Because of the salt water, some children experience an itchy head after the water dries, like what you might experience after swimming in a chlorinated pool or going to the beach. As such, we recommend washing your child's hair within a day or two after the visit. Does this sound okay?

b. **<u>Mom Response of 'No'</u>**: Okay, no worries. On the day of your visit, we can show you the cap, give you more information, and decide whether it makes sense to try on the EEG cap.

Spanish

1. **<u>Experimentador:</u>** Nos gustaría recolectar la actividad cerebral (EEG) de su hijo/a para observar cómo está desarrollando su cerebro. Para medir esta actividad cerebral, pondremos en la cabeza de su hijo/a un gorro suave y elástico diseñado especialmente para niños. El gorro que usaremos es seguro y no envía nada al cerebro de su hijo/a. No es necesario que decida si desea participar en la grabación del EEG en este momento. Le daremos más información sobre esta actividad durante su visita al laboratorio. Sin embargo, si usted cree que le gustaría participar en el EEG, me gustaría hacerle algunas preguntas sobre cómo peina generalmente el pelo de su hijo/a para entender cómo le puede quedar el gorro del EEG. ¿Le parece bien?
   a. **<u>Si la mamá responde 'Si'</u>**: ¿Cómo suele llevar el cabello su hijo/a o cómo es probable que lo lleve el día de su visita? (RA note: If examples are needed, you can say: ¿Cuál de las siguientes opciones describe mejor el peinado habitual de su hijo/a?)
      1. Pelo corto (rapado, ondas, Caesar-cut)
      2. Suelto
      3. En colas o en moños
         a. **<u>Si tiene colas o moños:</u>** Pregunta si la mamá puede evitar el uso de accesorios o quitárselos el día de la visita. Considere también enviar sugerencias de estilo.
      4. Trenzas (con su propio pelo o postizo)
         a. **Si tiene trenzas**: ¡Gracias por dejarme saber! Si prefieres, podemos intentar agendar la visita en un momento en el que su cabello esté suelto. O, podemos proceder con la visita con su cabello en ese estilo de peinado, ¿Qué le parece?
      5. Rastas
         a. **Si tiene rastas:** ¡Gracias por decirme, es bueno saberlo! Podemos asegurarnos de intentar trabajar alrededor de sus rastas para obtener la mejor medida o podemos hablar más sobre esto el día de su visita. ¿Qué le parece?
      6. Otro

a. ¿Cómo piensa usted que el gorro (parecido a un gorro para nadar o para bañarse) quedaría en la cabeza de su hijo/a con este estilo de peinado?

ii. **Si la mamá responde 'Si'**: ¿Hay algo más que usted desea que sepamos sobre el cabello de su hijo/hija?

iii. **Si la madre aún sigue interesada:** : Perfecto. Adicionalmente, el gorro de EEG se moja en agua un poco salada con un poco de champú para bebé. El agua no es muy salada, casi igual de salada que las lágrimas. Debido al agua salada, algunos niños experimentan comezón en su cabeza cuando el agua se seca, como lo que podría sentir después de nadar en una alberca con cloro o ir a la playa. Por lo tanto, recomendamos lavarle el cabello a su hijo/a uno o dos días después de su visita. ¿Le parece bien?

b. **Si la mamá responde 'No'**: Ok, no se preocupe. El día de su visita le podemos mostrar el gorro, darle más información, y decidir si le gustaría probar el gorro de EEG.

Supplement 3: EEG video in English
Available upon request to the corresponding author.

Supplement 4: EEG video in Spanish
Available upon request to the corresponding author.
